# Exploring public views on the use of synthetic datasets for research - results from the DELIMIT study

**DOI:** 10.23889/ijpds.v9i5.2925

**Published:** 2024-09-18

**Authors:** Anne-Marie Nybo Andersen, Siddartha Aradhya, Josephine Funck Bilsteen, Nick Bowden, Erin Early, Kathleen Falster, Martin Flatø, Rob French, Erika Hagemann, Katie Harron, Jen Keating, Gissler Mika, Rebecca Mitchell, Alicia Montgomerie, Nathan Nickell, Irene Papanicolas, Rhiannon Pilkington, Paul Romitti, Sujitha Ratnasingham, Lisa Smithers, Nieves Valdés, Ben Wilson

**Affiliations:** 1 Department of Public Health, University of Copenhagen, Denmark; 2 Stockholm University Demography Unit, Sweden; 3 University of Otago, New Zealand; 4 Ulster University, Northern Ireland; 5 School of Population Health, Faculty of Medicine and Health, University of New South Wales, Sydney, Australia; 6 Norwegian Institute of Public Health, Oslo, Norway; 7 School of Social Sciences, Cardiff University, Wales; 8 The University of Western Australia, Australia; 9 UCL, England; 10 Cardiff University, Wales; 11 THL Finnish Institute for Health and Welfare, Finland; 12 Australian Institute of Health Innovation, Macquarie University, Australia; 13 BetterStart Health and Development Research,School of Public Health, Faculty of Health and Medical Sciences, The University of Adelaide, Australia; 14 Max Rady College of Medicine, Department of Community Health Sciences, University of Manitoba, Canada; 15 Brown University, USA; 16 Iowa Registry for Congenital and Inherited Disorders, Department of Epidemiology, University of Iowa, USA; 17 ICES, University of Toronto, Canada; 18 School of Public Health, The University of Adelaide; 19 UAI, Chile; 20 Stockholm University Demography Unit, Sweden

## Objectives

To understand how we can better initiate and run international collaborations to deliver tangible and impactful findings using population-wide linked health and education data.

## Approach

Representatives from 14 countries (America, Australia, Canada, Chile, Denmark, England, Finland, Germany, New Zealand, Northern Ireland, Norway, Scotland, Sweden, Wales) summarised the potential for research using their linked administrative health and education data. The scope for collaboration and comparison of research findings across countries was explored.

## Results

Several substantive research themes emerged, including the quantifying of differences in educational outcomes by health conditions and other early life factors, using health data to better identify and explore the special educational needs identified in educational records, using linked health and education data to identify and explore reasons for absenteeism from school.

## Conclusions

The workshop showcased the primary themes for research and the data assets available within each country for comparative work. Further work is required to more robustly document the available detail within these datasets, collaboratively develop protocol templates for comparative studies, and develop pilot, proof of concept, studies.

